# Recording harms in randomised controlled trials of behaviour change interventions: a qualitative study of UK clinical trials units and NIHR trial investigators

**DOI:** 10.1186/s13063-024-07978-1

**Published:** 2024-03-04

**Authors:** Diana Papaioannou, Kirsty Sprange, Sienna Hamer-Kiwacz, Cara Mooney, Gwenllian Moody, Cindy Cooper

**Affiliations:** 1 Sheffield Clinical Trials Research Unit, Division of Population Health, School of Medicine and Population Health, University of Sheffield, Regent Court, 30 Regent Street, Sheffield, S1 4DA UK; 2 Nottingham Clinical Trials Unit, University of Nottingham, Nottingham, NG7 2RD UK; 3 Centre for Trials Research, Cardiff University, Neuadd Meirionnydd, Heath Park, Cardiff, UK

**Keywords:** Adverse events, Harms, Non-drug, Non-CTIMP, Behavioural interventions, Qualitative, Focus groups, Clinical trials, Clinical trials unit

## Abstract

**Background:**

Harms, also known as adverse events (AEs), are recorded and monitored in randomised controlled trials (RCTs) to ensure participants’ safety. Harms are recorded poorly or inconsistently in RCTs of Behaviour Change Interventions (BCI); however, limited guidance exists on how to record harms in BCI trials. This qualitative study explored experiences and perspectives from multi-disciplinary trial experts on recording harms in BCI trials.

**Methods:**

Data were collected through fifteen in-depth semi-structured qualitative interviews and three focus groups with thirty-two participants who work in the delivery and oversight of clinical trials. Participants included multi-disciplinary staff from eight CTUs, Chief investigators, and patient and public representatives. Interviews and focus group recordings were transcribed verbatim and thematic analysis was used to analyse the transcripts.

**Results:**

Five themes were identified, namely perception and understanding of harm, proportionate reporting and plausibility, the need for a multi-disciplinary approach, language of BCI harms and complex harms for complex interventions. Participants strongly believed harms should be recorded in BCI trials; however, making decisions on “how and what to record as harms” was difficult. Recording irrelevant harms placed a high burden on trial staff and participants, drained trial resources and was perceived as for little purpose. Participants believed proportionate recording was required that focused on events with a strong plausible link to the intervention. Multi-disciplinary trial team input was essential for identifying and collecting harms; however, this was difficult in practice due to lack of knowledge on harms from BCIs, lack of input or difference in opinion. The medical language of harms was recognised as a poor fit for BCI trial harms but was familiar and established within internal processes. Future guidance on this topic would be welcomed and could include summarised literature.

**Conclusions:**

Recording harms or adverse events in behaviour change intervention trials is complex and challenging; multi-disciplinary experts in trial design and implementation welcome forthcoming guidance on this topic. Issues include the high burden of recording irrelevant harms and use of definitions originally designed for drug trials. Proportionate recording of harms focused on events with a strong plausible link to the intervention and multi-disciplinary team input into decision making are essential.

**Supplementary Information:**

The online version contains supplementary material available at 10.1186/s13063-024-07978-1.

## Background

Harms, commonly referred to as adverse events (AEs), are recorded and monitored within randomised controlled trials (RCTs) to ensure the safety of trial participants; a core principle of the international ethical and scientific quality standard for clinical trials, ICH Good Clinical Practice (ICH-GCP) [1]. Serious harms or adverse events (SAEs), those resulting in death, hospitalisation, or a life-threatening episode for example, are reported in real time during the trial to the Sponsor. SAEs which were not expected from a trial intervention (known as SUSARs or RUSAEs) are reported in strict timelines to trial regulatory authorities. The accumulating unblinded data on harms is independently monitored by Data Monitoring Committees (DMC), who hold the responsibility to ensure the trial remains safe to continue. At the end of a trial, the evaluation examines both the benefits and the risks of the intervention.

It is therefore important that the data collected on harms is of high quality to ensure the risks of interventions are accurately evaluated throughout and at the end of a trial. However, in behaviour change interventions (BCI), which may include psychological therapies, lifestyle interventions and public health interventions, data on harms is recorded inconsistently within RCTs. A review of 151 BCI trial protocols found 15/151 (10%) stated they would *not* record non-serious harms, and 7/151 (5%) would *not* record serious harms. It was unclear from 41/151 (27%) and 55/151 (36%) trial protocols planned to record non-serious and serious harms, respectively. Little detail on the approaches or methods used to record harms were given [2].

The CONSORT harms extension [3] provides detailed recommendations on how to report harms within RCTs. However, the inconsistent methods and lack of clarity in recording harms in BCI trials suggests there may be uncertainty in the approach to use. There is no specific guidance on recording harms in BCI trials.

### What is a harm?

One problem may be the way in which harms are defined in BCI trials. The review of BCI protocols found 25% used ICH-GCP definitions to non-serious harm, whereas 52% provided no definition for harms [2]. No protocols defined harm as per the CONSORT harms definition: “a totality of the totality of possible adverse consequences of an intervention or therapy; they are the direct opposite of benefits, against which they must be compared” [4].

Literature exists which demonstrates potential unintended harms from BCIs, which may not necessarily be captured by the ICH-GCP definition [1]. For example, school-based social and emotional learning (SEL) interventions that targeted young people have identified participants felt negatively labelled and resulted in rejection of school, and being labelled as a “SEL” failure might enhance students’ status amongst peers [5]. In another trial, improvement of physical activity facilities in a deprived area resulted in participants feeling more marginalised and disadvantaged, exacerbated by the new facilities being used more by affluent outsiders [6]. There may also be a misconception that harm is not possible from BCIs, which may be given as a justification for not recording non-serious harms [2]. The CONSORT Social and Psychological Intervention extension notes the potential for unintended harms from BCIs [3].

### How to record harms efficiently?

There are also issues in how to record harms efficiently, particularly in populations who experience a large number of harms as part of their illness or other characteristics such as age. Trials may use a huge amount of resource and staff time in recording obviously irrelevant harms (not related to the intervention). This can be particularly pertinent in populations that experience a high frequency of harms, such as older adults [7] or those frequently hospitalised, e.g. substance abuse [8]. However, to make the decision to exclude irrelevant harms from recording, triallists need clarity on what harms may be plausibly caused by BCI. Drug trials have reference safety information, such as the Summary of Product characteristics or Investigator’s Brochure which summarise all possible harms that may be caused by drugs. There are no such documents for BCIs; instead triallists need to identify before a trial starts what harms are likely from their BCI being evaluated. In the review of BCI trial protocols, only 22/151 protocols listed expected harms in the protocol, and 4/22 described how they identified what harms might be possible from the BCI being evaluating [2].

This study was part of a wider project [9] that aimed to develop guidance on how to record harms in behaviour change intervention RCTs. The aim of this qualitative study was to explore the experience and perspectives of multi-disciplinary experts, including Patient and Public representatives (PPI), involved in designing and delivering BCI trials in relation to recording harms.

We aimed to identify the operational considerations for monitoring harms during trials. This included identifying methods or approaches for recording harms which have worked, or conversely which have not worked.

## Methods

### Approach and rationale

We used a phenomenological approach in that we aimed to investigate the experiences and understanding of an issue [10]. Our approach is pragmatic, rather than theory building or testing, in that we hoped to gain an understanding of the current practice of recording harms in BCI trials to allow insight into how decisions are made on recording harms, and whether the approaches taken were beneficial or problematic.

Individual and paired qualitative interviews were conducted as well as focus groups (FG). Paired interviews were undertaken with participants from the same organisation, at their request, to facilitate sharing of ideas and experiences. Focus groups were conducted with participants who had similar expertise (e.g. trial manager or PPI representatives), to allow sharing of ideas and experiences, clarification of views in relation to others and exploration of differing or similar opinions [11]. Data from the interviews and focus groups were merged for completeness of findings [12].

### Ethical approval

Ethics approval for the research was received from the Research Ethics Committee (REC) of the School of Health and Related Research (ScHARR) at the University of Sheffield on 28th January 2022 (ethical approval ref: 044669).

### Researcher characteristics and context

The research team consisted of four female researchers from the Universities of Sheffield and Nottingham (KS, DP, SHK and CM) who have an interest in improving recording of harms in behaviour change trials for efficiency and relevance to the context of behaviour change.

KS (MSc, B.A, BSc) is an Assistant Professor at Nottingham Clinical Trials Unit, who has extensive experience in conducting qualitative interviews and focus groups [13–16]. DP (MSc, BChD) is an Assistant Director at Sheffield Clinical Trials Research Unit with experience in designing and implementing trials, and safety reporting. SHK (MSc, BSc) is a Research Assistant at Sheffield CTRU. CM (MSc, BSc) is a Senior Trial Manager at Sheffield CTRU with experience in conducting focus groups. KS provided training to SHK and DP in qualitative interviewing and focus groups; this included KS observing and providing feedback on technique prior to solo-interviewing or FG facilitation by SHK or DP.

The interviews were mostly conducted with individuals unknown to the interviewers/FG facilitators. Of the 32 participants, two were known to the researcher (KS) conducting the interview and three were known to CM in a pilot focus group with colleagues at the Sheffield CTRU to test the FG topic guide (Data from the pilot FG are included in the analysis). Although we recognise the risk for power imbalance or social desirability bias, we deem this to be low. The two individuals known to KS were through a previous working relationship. The three participants known to CM had a working relationship with her at the time of interview and continue to do so. However, at the time of interview, there was no hierarchy within this working relationship.

Participants were told that the interviews and focus groups were part of a wider project to inform future recommendations or guidance in how to record harms in BCI trials.

### Sample

A purposive sample [17] of trial managers, chief and principal investigators (included clinicians as well as behaviour change experts), data collectors, Sponsors, Quality Assurance staff, statisticians, oversight committee members and patient and public representatives were recruited. We focused on recruiting those who had experience in designing and delivering BCI trials. Focus groups were conducted with similar groups of individuals for example, patient and public representatives or trial managers. Individuals were identified to take part in the study in a number of ways. This included:

(i) Review of funded research through the National Institute for Health and Care Research (NIHR) Funding and Awards Website by reviewing staff listed in protocols of relevant completed or in progress trials. Twenty-eight studies were identified from the NIHR funding Awards website and reviewed by the Project Steering Committee (PSC) for omissions and to assist with the selection of the studies. Seven studies were purposefully selected with the help of the PSC, to represent a range of geographical locations in the UK, CTUs and trials involving a range of behaviour change interventions. Trials were identified from the NIHR Funding and Awards database or by browsing trials on UK CRC CTUs’ websites. An invitation email was sent to eight individuals: six accepted and two declined.
(ii) An expression of interest to take part was emailed to the UK Trial Managers’ Network (UKTMN) distribution list and posted on Sheffield CTRU Twitter page (UK CRN agreed to retweet). This notice asked for trial managers and researchers who wanted to take part to describe their experience in BCI trials, with a short description of the trials they had been involved in (i.e. type of intervention, population). Twelve individuals responded expressing an interest to take part; ten were sent an invitation to take part; nine accepted and one participant did not respond to the invite. The two participants who were not invited had (by their own admission) no or very little experience in delivering BCI trials.
(iii) Project Steering committee members and Sheffield CTRU colleagues provided relevant contacts; authors from the literature included in the scoping review conducted as part of the wider project were identified. Ten individuals were invited; 8 accepted and 2 did not respond.

An established PPI group (the Deep End Yorkshire and Humber Patient and Public Involvement Group) with experience in advising on clinical trial design, were known to the Sheffield researchers (DP, CC, CM, SHK) and were approached to take part in a PPI focus group (*n* = 6). A pilot focus group of Sheffield CTRU staff (*n* = 3) was undertaken.

### Recruitment and data collection

Potential participants who had indicated an interest in taking part were sent an invitation email including a participant information sheet (see Additional file 1) describing the purpose of the study, and a consent form (see Additional file 2).

Interviews were conducted remotely using an online platform (Google Meet) and were audio recorded, with consent, to allow transcription and analysis. (The video recording function in Google Meet was not used). Two focus groups were conducted remotely, including a pilot focus group at Sheffield CTRU (CM and SHK) to test the topic guide. The use of both interviews and focus groups (FGs) for data collection was to maximise time and resources and to enable participation. FGs, although more challenging to schedule, offer participants the opportunity to identify and clarify views in relation to others with similar lived experience. We did not anticipate the data generated from either method to differ widely, and on familiarisation with the data during the analysis we were correct in our assumption. The data was therefore treated as one dataset for the analysis.

The Patient and Public focus group was carried out face-to-face by three researchers (DP, KS and SHK); also present was Dr Kate Fryer who facilitates the PPI group. Field notes were also taken during interviews and focus groups. Interviews ranged in duration from 26 min to 1 h 5 min; the focus groups duration ranged from 1 h 5 min to 1 h 12 min.

A structured topic guide was developed to help focus discussion (Additional file 3); the first draft of the topic guide was developed using previous literature to inform the core topics and then reviewed by the study team (DP, KS, SHK, CM) and the Project Steering committee. Participants were provided with a summary of literature 1 to 2 weeks prior to their interview or focus group. The literature summary included general recommendations and proposed methods for recording harms in BCI trials, as well as suggested mechanisms and categories of harms identified from a scoping review [18] undertaken as part of the wider project [9]. The intention of sharing the literature summary was firstly to understand whether this literature was known to participants, and to stimulate discussion on its usefulness and the practical implications on recording harms in BCI trials. We provided this literature in advance of the interviews/FGs to give participants “thinking time”, and so that they were aware of potential harms possible from BCIs that may fall outside their current beliefs of what constitutes harm in the context of a BCI trial.

### Analysis

The ScHARR Transcribers Group transcribed the audiorecorded data for the interviews and focus groups. Transcripts were anonymised by using non-identifiable codes and removing identifying information. Respondent or member validation was not conducted; however, transcript validation was undertaken by a member of the team (SHK or KS) by reviewing the transcript against the audio recording for accuracy.

Thematic analysis, using an inductive/deductive approach [19, 20], was used to identify participants’ perspectives regarding harms recording in RCTs. Themes were identified from the interview schedule, as well as exploration of themes emerging from the data. KS, SHK and DP double coded three transcripts to check interpretation of the findings and to produce an initial draft coding framework, and to increase the reliability of the research [21]. Differences were resolved by discussion between KS, SHK and DP as required. The remaining transcripts were then divided equally between KS and SHK and coded against the draft framework. Categories and themes were developed by constantly refining the coding scheme with regular discussion between the two coders (KS and SHK) to agree the final framework. Master and subthemes were identified. Data from the interviews and focus groups was analysed as one data set.

Microsoft Office Excel was used to manage the data which was readily available across the researcher sites. Data saturation was monitored during the process of data collection and considered no new themes were emerging by the end of the process.

This study followed the consolidated criteria for reporting qualitative research (COREQ) guidelines [22] (see Additional files 4 and 5).

### Findings

Fifteen in-depth semi-structured qualitative interviews (11 individual and 4 paired interviews) and three focus groups were undertaken between February and May 2022. Thirty-two multi-disciplinary experts who design and deliver trials took part and included oversight committee members, Patient and Public representatives, Chief Investigators, Trial managers, Data Collectors, Sponsors and Statisticians. Interviewees represented ten areas in the UK including Sheffield, Nottingham, York, London, Cardiff, Loughborough, Leeds, Hertfordshire, Bristol, and Exeter. Eight UK CTUs were represented in the study. Table 1 summarises the characteristics of participants.

**Table 1:** Focus group and Interviewee characteristics

Participant characteristics^a^	Interviews^b^	Focus groups ^c^
Trial managers	3	**7**
Chief or principal investigators	5	
Data collectors	**2**	
Quality assurance staff	**2**	
Statisticians	**2**	
CTU Directors or other senior staff	**3**	
Sponsors	2	
Patient and public representatives		6

^a^Individuals are counted under their main role or the role they identified however some had experience across multiple roles, e.g. Chief Investigator and oversight committee member

^b^Ten interviews conducted by KS with SHK; 3 interviews conducted by SHK; 2 interviews conducted with DP and SHK; 11 were individual interviews and 4 were paired interviews

^c^CM conducted one pilot focus groups with Sheffield CTRU trial managers; DP, KS and SHK conducted the PPI focus group; DP and SHK conducted one trial manager focus group

### Themes

Five broad themes emerged from the analysis of the interview and FG transcripts:

1. Perception and understanding of harms
2. Proportionate reporting and plausibility
3. The need for a multi-disciplinary approach
4. Language of BCI harms
5. Complex harms for complex interventions

Table 2 summarises the findings from the themes and subthemes.

**Table 2:** Overview of key findings by theme and subtheme

*Subthemes*	*Summary of key findings*
***Theme 1: Perception and understanding of harms***	
Importance of harms recording	• Essential for BCI trials • Range of opinion on risk level; seen as both low risk (because not a CTIMP) or high risk (harms unknown therefore risky) • Important to patient and public representatives
Perceptions of what is a harm	• Largely informed by individual experience, knowledge of clinical trials and colleague influence due to the lack of guidance. • Greater understanding on what constitutes a Serious Adverse event in BCI trials; confusion around what constitutes a non-serious harm or AE. • Safeguarding/AEs/SAEs- lack of distinction and overlap • Participants found harm difficult to quantify and define in BCIs and most agreed there was a subjective nature to harms in BCI trials, i.e. what might be considered harmful to one person might be different to another.
Factors influencing harms	• Study population • Intervention; established or novel • Plausibility of being related to the intervention • Outcomes already being collected within the trial which may capture harms • Meaningfulness to patients • Importance of collecting different perspectives on harms (family member, significant other) as well as the trial participant.
Approaches to harms recording	• Majority applied the ICH-GCP definitions for harms, AEs and SAEs given they are responsible and standard in clinical trials • Importance of embedded qualitative research routinely asking for harms (currently few participants report qualitative research did this). • Importance of feasibility and pilot studies capturing harms
Awareness of literature	• Lack of awareness about the literature on harms in BCIs • Most participants viewed a literature summary on categories and mechanisms of harms, methods, and recommendations as useful to stimulate discussion amongst trial teams around harms from an intervention.
***Theme 2: Proportionate reporting and plausibility***	
Burden of recording harms	• Most participants perceived a lack of pragmatism on determining the events to record as harms, with over-reporting of harms in BCI trials unrelated to the intervention. • Large burden of reporting on trial staff and trial participants • Data wastage and difficulty in finding signals in the data.
Plausibility	• A proportionate approach should be taken to avoid wasting resources and improve data quality. • Plausibility is the key factor for a proportionate approach, there must be a plausible link that the intervention could cause the harm.
***Theme 3: The need for a multi-disciplinary approach***	
	• Essential for multi-disciplinary team input; variety of perspectives required on recording harms. • Lack of knowledge or understanding of harms from BCIs or lack of input could make decision making difficult. • Difference in opinion and conflict on recording harms decisions. • Trial manager burdened with responsibility of making decisions.
***Theme 4: Language of BCI harms***	
Medical language	• Lack of suitable alternative definition for harm, therefore ICH-GCP used • Led to highly medicalised language which was deemed inappropriate in context of BCI trials. • SAEs easier to identify than AEs. • New harms language for BCIs is considered essential.
***Theme 5: Complex harms for complex interventions***	
Causality	• Driven by direct contact with the participant, requiring contextual information and therefore resources • Case-by-case decisions on attribution of a harm to the intervention. • Participants reported instances where it was not appropriate to keep asking for further information to determine causality of an intervention where an event or data may be sensitive.
Future guidance	• Future guidance would be welcomed, particularly practical application for e.g. protocol template wording. • Direction and endorsement or support from regulatory bodies like the Heath Research Authority was also sought. • Providing a summary of relevant literature in an accessible document was thought useful. • Details on basic theorising of harms from interventions was requested. • Difficulty in writing guidance was noted because it was unlikely to be a one size fits all, but generic guidance may not be useful. • Guidance on recording hams would be another guidance document to follow and ‘another thing to do’ within limited trial resources.

*CTIMP* Clinical Trial of Investigational Medicinal Product

## Theme 1: Perception and understanding of harms

### Importance of harms recording

Recording and reporting of harms in behavioural change interventions (BCIs) was agreed to be just as important as for any other type of intervention with patient safety, ethics and undertaking unbiased assessments highlighted as key factors, despite many viewing BCIs as low risk.

“We need to do it, it’s an important part of running a trial and assessing the merits of an intervention… you can’t assess an intervention unless you assess its merits and its harms… we are supposed to be in equipoise, right? So, in not assessing harms, or at least not assessing whether you need to assess harms, it’s not equipoise, it’s biased” (P2, Interview, Chief Investigator)

Opinions ranged from being seen as low risk compared to other interventions such as CTIMPs, to high risk in that harms could be unknown and therefore missed.

“…subject of adverse events came up and you know, it’s really a very low risk study and we did not, we don’t expect there to be any adverse events because it’s not, the intervention is not of that nature, but we did say we would monitor any incidents of concern…” (P8, Paired Interview/Trial Managers)

Recording harms in BCI trials was important to the patient and public representatives who did not view harms recording in BCI trials as that dissimilar from drug trials.

“You’re injecting a thought process, in a different way…you’ve gone and you’ve been injected mentally with a thought process that has affected you. But what’s the physical measurement of that injection of a thought process?” (P28, Focus group, Patient and public representative)

### Perceptions of what is a harm

The paucity of any approved guidance meant perception of harms in BCIs was informed largely by individual experience, knowledge of clinical trials and colleague influence. A common issue trial teams struggled with was confusion around what could be considered a non-serious adverse event, with greater understanding of what constituted an SAE in BCIs. Several CTU staff and Investigators commented on the potential for lack of clarity or overlap between safeguarding and harms or AEs.

“So, when does it then become an adverse event? I think we’re still left with that…even if certainly the team who are the researchers in this, you know, they do have a very good knowledge of what harms are and deal with them very much on a day-to-day basis and, and how, what impact these have on, on the people around them.” (P10, Paired Interview, Programme/Trial Manager)

All participants found harm difficult to quantify and define in BCI trials and PPI representatives noted the issue of harms being subjective from person to person.

“We’re all very, very different even in this room now, and if we all took part in a, a softer type trial [behaviour change trial]…we would all be very different, culture, age, sex, colour, whatever, we would all react differently…and it’s very soft to measure, isn’t it? I think that’s the difficulty.” (P28, Focus group, Patient and public representative)

### Factors influencing harms

Several factors were identified as influencing the presence and prevalence of harms. This included study population, the intervention(s) and whether it was established or novel, plausibility, outcomes, and meaningfulness to patients.

The importance of collecting harms from different perspectives and viewpoints was important to the PPI representatives, both in terms of collecting harms that were experienced by the trial participant, but also harms experienced by other individuals than the trial participant.

“…if you only base the recording of your harm on the individual, you only get part of it, you don’t get all of it. But if you include the significant others around that person, you might get the bigger picture.” (P27, Focus group, Patient and public representative)

### Approaches to harms recording

Approaches to identifying, recording, and reporting across trials were inconsistent; however, most participants applied the ICH-GCP definitions of AEs and SAEs [1]. Although the majority of participants saw ICH-GCP definitions as a problematic fit for BCIs, it was also seen as a recognisable and standard for use in trials and trial reporting.

“…we wanted something that people would acknowledge and recognise. So, we felt we were comfortable with that because it sits with how we’re trained, with the GCP…” (P2, Interview, Chief Investigator)

Several trial staff participants noted the importance of embedded qualitative research but many acknowledged that although they routinely included qualitative methods within their trials, it rarely explored harms from an intervention.

“I think qualitative data is quite important for catching the whole experience of what happened during the intervention, whereas these adverse event forms are black and white, this happened and you don’t get that rich data that you do through the interviews” (P25, Interview, Trial Coordinator)

A few participants noted the importance of undertaking this work early on within intervention development work, with an expectation that key harms would emerge in feasibility or pilot studies.

“…we would expect anything that was going to come up to have come up in those feasibility and pilot studies”. (P7, Interview, CTU Director)

### Awareness of literature

Although there was a general awareness of AEs and SAEs in trials, there was a lack of awareness of the literature on harms in BCIs amongst CTU Staff and Trial Investigators.

“…we do assess adverse events but looking at all the literature I wonder whether we’re looking at enough…” (P7, Interview, CTU Director)

The literature summary, which described categories and mechanisms of harms, as well as recommendations and methods on how to record harms in BCIs, was viewed by most participants as useful and could stimulate discussion amongst trial teams to identify potential harms from an intervention.

“I would probably have a look at these models [The literature summary] for when, before we go through the adverse events and encourage the team to do the same and have a discussion on that” (P6, Interview, Chief Investigator)

## Theme 2: Proportionate reporting and plausibility

Recording harms was cited for many reasons, namely DMC oversight to aid decision making on trial continuation, patient safety monitoring, to evaluate effectiveness of an intervention (between group comparison), trial transparency and as a requirement for some journal publications.

### Burden of recording harms

Whilst a few CTU staff and trial investigators reported a level of pragmatism to determine what events to record as harms within a BCI trial, a more common view was a perceived lack of pragmatism and a belief that there was a tendency to over report harms in BCI trials for little purpose or benefit. Participants thought this was disproportionate to the risk of intervention and reflected the events which occurred in the everyday life of trial participants rather than harms likely to be caused by an intervention.

“…you know, life is hard and things are gonna happen to people – what’s really important? Like when you get all of your data at the end of the study and you have this tons and tons of harm data, what are you going to do with it? What’s it going to inform?” (P12, Focus Group, Trial Manager)

This was particularly pertinent in populations where high frequency and clearly unrelated adverse events occurred for, e.g. older adults, (an issue also relevant to non-BCI trials).

“We had to collect all of these things that had happened to them, and really what we got was an account of people’s lives day to day, post 65 and the illnesses and adverse events that can just overtake people.” (P23, Interview, Chief Investigator)

Sponsors and CTU staff were concerned about the impact over-reporting had, namely the burden of reporting for trial staff as well as the trial participant, resulting in fatigue for recording harms.

“My colleagues who do it [AE collecting], dread them” (P26, Interview, Sponsor Representative / Research Governance Manager)

A further concern was voiced around perceived data wastage and difficulty in finding signals within the data.

“so there’s a whole lot of these adverse events that have nothing to do with treatment so even trying to pick out any treatment related ones which are the ones you’re only really interested in, you drown in the noise” (P20, Interview, CTU Director/Statistician)

### Plausibility

All participants believed that a proportionate approach should be taken to avoid wasting resources, improve data quality and reduce burden on both participants and staff. Plausibility was seen as the key factor by several participants in determining a proportionate approach to recording harms, that is there had to be a plausible link that the intervention could cause the harm.

“…it’s got to be about plausibility…separating that plausibility because of the intervention from something that would be very likely to happen anyway” (P9, Interview, Sponsor Representative / Research Governance Manager)

## Theme 3: The need for a multi-disciplinary approach

Multi-disciplinary input from relevant stakeholders was considered essential to the process of identifying potential harms from an intervention, and in agreeing how to record and report these. This included academic, clinical, and trial management expertise as well as PPI engagement, alongside validation from QA, Sponsors, ethics, and oversight committees.

Different perspectives were valued by participants, particularly PPI input.

“So, our service users, carers and clinicians, are absolutely instrumental in the design of the intervention but also in the development of the feasibility trial. So, you know, they too would feed into that process in terms of harm reporting and if there’s anything that we might have missed…” (P13, Focus group, Trial Manager)

Making decisions on how and what to record as harms in BCI trials was often difficult. Trial managers described issues such as a lack of knowledge and understanding of harms from BCIs to confidently identify them or a lack of input or opinion from some trial team members. Several participants described differences in opinion amongst multi-disciplinary trials teams, which underlined the importance of including a range of viewpoints.

“…it becomes a bit of a battle of wills about who’s your loudest investigator, sometimes, as to how much you end up reporting.” (P12, Focus group, Trial Manager)

“…I’m not sure how interested CIs would be in spending a lot of time looking at different frameworks to think about potential harms that there might be. In fact, they probably don’t, in a way, they don’t want to find any harms. So, it’s sort of – it’s good to have other stakeholders involved…” (P5, Focus group, Trial Manager)

Furthermore, one trial manager noted they had reluctantly taken on the responsibility of deciding the events to record as harms.

“But some of the behavioural studies don’t have a huge clinical involvement, necessarily, so it’s kind of – sometimes I feel like, as a trial manager, everyone just leaves me to do decide things. And I don’t want to decide things.” (P12, Focus group, Trial Manager)

## Theme 4: Language of BCI harms

The language around harms was highly medicalised, being predominantly ICH-GCP informed, and was therefore deemed inappropriate for use with all BCIs. However, ICH-GCP definitions and processes were typically applied to BCIs due to the lack of any other suitable alternative, the subjectivity of what harm is in BCIs, the requirement to follow local SOPs and because these definitions were universally understood.

“it’s modelled on obviously pharma-trials, so we’ve got this mismatch between how we collect the data on potential harms and how it’s interpreted’… ‘it doesn’t really match behaviour change trials, it hasn’t got that flexibility” (P23, Interview, Chief Investigator)

Despite the medicalised language of SAEs, they were generally deemed simpler to identify in BCIs than AEs, but no clear process on how to do this was described. Management of SAEs in BCIs typically followed local SOPs using the ICH-GCP guidance and timelines on urgent reporting.

A new harms language for BCIs and definitions of harms equivalent to AEs and SAEs was considered essential by both trial staff and PPI representatives. Clarity was also sought in the differences between AEs, SAEs, safeguarding and compliance in BCIs.

“I think that you need a whole new language around it, ditch SAEs and think of something else because that would take people down that route … it’s not the right word is it” (P23, Interview, Chief Investigator)

“…you guys should have a working definition, not a definitive definition, a working definition of what is harm, and that working definition should encompass everything you think is harm. With time that definition will be refined, and it will be standardised, okay, that is how everything has been defined in the scientific and the social sciences world.” (P27, Focus group, Patient and public representative)

## Theme 5: Complex harms for complex interventions

Complex interventions and complex populations were felt to equate to more complex harms; therefore, data collection for BCI harms was considered more complex due to these factors. As mentioned within the *Perception of harm* subtheme, harms were considered subjective which contributed to the complexity.

### Causality

Identification of harms and allocation of causality was more likely to be driven by direct contact with the participant. For example, data were more likely collected via contextual information gathering thereby requiring participant co-operation and more staff resource. This meant decisions were generally made on a case-by-case basis rather than using pre-defined limits or event descriptors. The quality of data is therefore dependent on information which may or may not be adequate to assign causality with confidence. Trial staff reported often data was too sensitive to keep asking for to gather more info to assess harms.

“I know that judgement call is not scientific, well it’s borne of experience but it’s not following a, you know, we’ve tried to put together a tree and come unstuck…….…[Do] we have enough information that we are confident that we can make a judgement call on that.” (P10, Paired interview, Programme/Trial Manager)

### Future guidance

The complexity of future guidance was discussed. Several participants felt greater consistency and guidance was specifically needed on how to record harms in BCI trials. Greater direction was also requested from authorities such as the HRA and funders as well as locally in units and co-ordinating centres (Quality Assurance systems).

Direction was sought beyond guidance or signposting, but offering practical application, such as wording and definitions for protocol templates. However, this was accepted as challenging due to the diverse and complex nature of BCIs.

“There’s no actual “this is what you need to go and do” and I think in a way you can’t because everyone’s studies are so different. Interventions are different, populations are different. So, having guidance that is generic enough to mean something I think is quite a challenge.” (P21, Interview, Trial Manager)

“I don’t think there’s a simple answer, is there? I think it’s very much dependent on the intervention and the population. I don’t think there’s gonna be a one size fits all.” (P4, Focus group, Trial Manager)

A summation of the literature on harms in BCIs into an accessible guidance document was proposed, basic theorising on harms possible, and training on harms in BCIs for trial team to better improve perception of harms as well as more practically in the recording and reporting processes.

“existing literature is so powerful… to review specific literature and your type of intervention to get specific about how your intervention could potentially be harmful”… “be a bit more flexible and broad in what literature you review first” (P19, Interview, Chief Investigator)

However, one Principal Investigator voiced a concern that whilst guidance would be welcomed, it may create new burden on trialists.

“…there’s a thousand and one things to do when you’re setting up a trial and whenever something new like this comes along, it never displaces anything else so there’s just one more thing to do and while I am supportive of it, I imagine it’s the kind of thing that people will say is a great idea at the beginning and then actually we can’t, we’ll either do a bad job of it or we won’t get round to doing it.” (P22, Interview, Principal Investigator)

## Discussion

The CTU staff and Trial Investigators who participated in this study were experienced in delivering and designing behaviour change interventions trials, yet they found making decisions on the ‘how and what to record as harms’ in these kinds of trials challenging and confusing. Approaches described for identifying, recording, and reporting harms across trials were inconsistent; however, most participants applied the ICH-GCP definitions of AEs and SAEs, and this is not surprising given the lack of alternative guidance for BCIs.

The CTU staff, researchers and patient and public representatives who participated in this study strongly believed harms should be recorded within BCI trials, for unbiased and thorough intervention evaluations. However, CTU staff perceived a significant burden on both trial staff and participants in recording large numbers of potentially irrelevant harms, which in turn drained trial resources and appeared to be for little purpose. Others have commented on the burden of recording harms in non-drug trials. In particular, where medical events are frequent in trial populations and unlikely to be caused by a behaviour change intervention, trialists and trial participants have spent large amounts of time recording unrelated harmful events ultimately irrelevant to the intervention evaluation [7, 8]. Assessment of causality of harms on an individual event level, i.e. determining if a harmful event is caused by an intervention, is also recognised in the literature as difficult if not impossible [23, 24] in behaviour change trials; this was a belief shared by participants in this study.

There was recognition that it was essential to receive input from the multi-disciplinary trial team when making decisions on the identification and collection for recording harms in BCI trials. PPI engagement is essential to understand potential harms and their importance. However, in practice, participants told us input from trial staff was not always forthcoming due to a lack of knowledge of harms from BCIs, difference in opinion on recording harms or a lack of input from team members. The responsibility for decision making was unclear, with concerns raised that the trial manager may be solely tasked with deciding on the approach to recording harms.

All participants agreed an emphasis on proportionate recording was appropriate, and that this could focus on events which had a strong plausible link to being caused by the intervention. However, there is a lack of guidance to inform researchers in the approach to take to record harms in BCI trials.

All agreed that the medical language and approach to recording harms, whilst required by regulatory authorities, did not seem to translate to BCIs. However, it was typically used due to its familiarity and perceived credibility as part of established organisational standard operating processes, and in the absence of alternative definitions or guidance. A new harms language appears to be required, with a broader universal definition for adverse events so that it reflects all trial interventions, not just drug interventions [25]. Examples in the literature demonstrate harms possible from BCIs that may not be captured by ICH-GCP definitions. For example, a peer support intervention in inflammatory bowel disease identified participants disliked ‘undesirable reactions’ such as pity or overreaction, being confronted with unwanted information. The intervention resulted in some participants feeling more anxious about their health where they were confronted with a possible negative future, which in turn led to social isolation, feelings of being weak or not control or dominated by their illness [26].

The CONSORT harms extension defines harm as “the totality of possible adverse consequences of an intervention or therapy; they are the direct opposite of benefits, against which they must be compared” [4]. There may be uncertainty around what constitutes an “adverse consequence” or difficulty in thinking what adverse consequences are possible. Categories and mechanisms of harms proposed in the literature, alongside examples of harms seen within BCIs, may help stimulate discussion amongst triallists, and be reviewed to determine their relevance to a BCI being evaluated. Our wider project [9] involved a scoping review which has collated this literature [18].

Case studies have been put forward that describe the approach to recording harms on specific behaviour change or public health trials [27–29], and a method “the Dark Logic model” [30] describes how to identify harms plausible from an intervention and is recommended by the CONSORT Social and Psychological Interventions extension [3].

However, this literature appeared little known amongst participants. CTU Staff and trial investigators would welcome guidance that includes a summary of relevant literature such as categories and mechanisms of harms from BCIs. However, future guidance was recognised as challenging with no one size fits all and seen as another task for trial teams to consider during busy protocol writing periods.

### Strengths and limitations

To our knowledge, this is the first study to ask multi-disciplinary trial staff and patient and public representatives about their views and perceptions on recording harms in BCI trials. Strengths of this study include the approaches used to maintain methodological rigour and trustworthiness. First, this is a novel study which explores the experiences of trial investigators in recording harms in behaviour change intervention trials. Second, this study achieved a broad understanding on this topic by involving multiple stakeholders across multiple disciplines, including the patient and public voice. Third, two independent researchers (KS, SHK) conducted the coding with emerging codes discussed between the two researchers and a third team member (DP), to ensure proper interpretation of the data and reliability of the results.

One limitation is the limited number of interviews conducted with harms data collectors, e.g. research nurses. However, we believe we achieved data saturation, with no new themes emerging at the end of the data collection stage. In addition, the other staff groups were well placed to comment on their perceptions on the experience of data collectors, which was evident in the study findings with regard to burden of harms recording. We do not believe new themes would have arisen from further interviews with the data collector staff group.

Another limitation was the use of the literature summary which was shared with participants prior to the interviews or focus groups taking place. This may have had the effect to bias opinions through prior knowledge. However, approximately 50% of participants viewed the summary before their interview/FG, with 50% using time within the interview/FG to read the summary. We did not discern differences in the views expressed between those who had or had not received the literature summary in advance of their interview/FG. The literature summary was very well received by most participants who found it helpful to consider potential harms from BCIs in the context of this literature; a key finding in our study was that this literature was not well known.

### Implications for practice

Findings from this qualitative study and the scoping review has been triangulated to develop recommendations on recording harms in behaviour change intervention trials. Online workshops with multi-disciplinary trial staff reviewed the draft recommendations, which will be finalised and published separately.

Most participants were unaware of the literature on categories, mechanisms, and examples of harms from behaviour change interventions that were identified in a systematic scoping review [18] conducted as part of the wider project; however, this literature was identified as helpful for trial teams to consider and stimulate discussion on recording harms in behaviour change intervention trials.

## Conclusions

The study findings, alongside a scoping review, have been used to develop recommendations for recording harms in BCI trials in forthcoming guidance. Recording harms or adverse events in behaviour change intervention trials is complex and challenging. Issues include (1) a high burden of recording irrelevant harms not related to an intervention, leading to wasting of resources and data wastage; (2) medicalised language and use of definitions originally designed for drug trials, which may not be wholly appropriate in the context of behaviour change. Proportionate recording of harms that focuses on events having a strong plausible link to an intervention, as well as multi-disciplinary team input into decision making rather than placing the responsibility on one team member such as the trial manager, was deemed essential. The CTU staff and investigators who participated were experienced in designing and delivering trials, and whilst they strongly believed harms should be recorded in BCI trials, they struggled with how to identify and collect harms.

### Supplementary Information

**Additional file 1.** Participant Info Sheet. This file provides the participant information sheet used for interview participants. It was adapted for the participants taking part in the focus groups.

**Additional file 2.** Informed Consent form. This file provides informed consent form used for interview participants. It was adapted for the participants taking part in the focus groups.

**Additional file 3.** Topic guide. This file provides the topic guide used in the interviews and focus groups.

**Additional file 4.** COREQ_Checklist_RHABITqualstudy. This file provides the completed COREQ checklist for the study.

**Additional file 5.** Coding tree-framework. This file provides the final coding tree/framework which was applied to the data in the transcripts.

## Data Availability

Data are available on reasonable request. The unpublished data used and/or analysed during the current study are available from the corresponding author on reasonable request.

## Abbreviations

AEs: Adverse events
BCI: Behaviour change intervention
CTU: Clinical trials unit
FG: Focus group
ICH-GCP: International Conference on Harmonisation GCP Guideline
NIHR: National Institute for Health and Care Research
PPI: Patient and Public Involvement
PSC: Project Steering Committee
RCTs: Randomised controlled trials
SAEs: Serious adverse events
SEL: Social and Emotional learning
UK CRC: UK Clinical Research collaboration
