# Supplementary material for: Recording harms in randomised controlled trials of behaviour change interventions: a qualitative study of UK clinical trials units and NIHR trial investigators
Source: Trials. 2024 Mar 4;25:163. doi: 10.1186/s13063-024-07978-1 (PMC10910772; doi:10.1186/s13063-024-07978-1)
Supplement: Supplementary file 1 — Additional file 1. Participant Info Sheet. This file provides the participant information sheet used for interview participants. It was adapted for the participants taking part in the focus groups. [file 13063_2024_7978_MOESM1_ESM.docx]

*Additional File 1 provides the Participant Information Sheet for individuals invited to an interview in the qualitative study as part of the RHABIT project. The Participant Information Sheet was modified for the focus groups conducted, for example by replacing ‘Interview’ for ‘Focus Group’, explaining the focus group duration was expected to be 90 minutes and reminding Focus group participants not to share what other participants discussed outside of the focus group.*

**Participant Information Sheet**

**Study title: Recordings HArms in Behavioural change Intervention Trials**

We would like to invite you to take part in an interview to talk about harms (i.e. adverse events/unintended consequences) recording in behaviour change intervention trials.

The information in the box below has been provided to give you an indication of what we will discuss in the interview.

Definitions:

- ‘Behavioural Change Intervention’ (BCI) = any intervention which intends to modify behaviour for e.g. psychological therapies, public health interventions, peer or social support, health communication or environmental policies, social and emotional learning
- ‘Harms’ = an unintended/unexpected consequence resulting from a BCI that is harmful to the individual. Trials often term harms as adverse events, which are often defined in medical terminology (“an untoward medical occurrence”)

Question examples:

- How to identify and assess harms in BCI trials?
- What recording processes are used and how do these work in practice?
- What knowledge/experience is there of existing models and typologies of recording harms in BCIs.
- What are the key considerations for researchers and future trials?

**What is the purpose of the RHABIT project?**

In drug trials, researchers will record and report the medical harms that patients may experience, for example, if they become very unwell or have to go to hospital. In trials of interventions which aim to change behaviours (like stopping smoking or eating more healthily) it is much less clear on which harms to record and how to report them. Researchers may miss harms that are important to patients or unnecessarily spend time measuring what is not important.

We have found behavioural change intervention trials record harms in different ways or not all. It is hard for researchers to know what they should record and to identify where participants may be being harmed.

The Recordings HArms in Behavioural change Intervention Trials (RHABIT) project aims to produce guidance on how to record harms in behavioural change intervention trials. This will hopefully improve the efficiency of harms recording, as well as consistency and transparency.

**What does the interview involve?**

We will invite people who are involved in running trials to attend a one-to-one interview to find out about problems they have had in harms recording, and what they have done to solve them. The information we find from reviewing relevant literature and interviews will be brought together to develop guidance. We will ask experts in clinical trials to see if they agree with these suggestions and write some recommendations**.**

If you agree to participate, you will be asked to attend a one-to-one online interview.

*Individual interview*

The purpose of these interviews is to explore participant's views on how harms should be recorded and reported in behavioural change intervention trials so that important events are not missed, or unnecessary things are not reported. The interview will be conducted either over the telephone or a media platform such as Google Meet, will be audio recorded and will last approximately 30 to 60 minutes.

You will be given some information on the topics to be discussed as well as a brief summary of the literature which you might find helpful to familiarise yourself with in advance of your scheduled interview.

The interview questions will be open-ended, to allow you the chance to raise the issues that you feel are important.

**Do I have to take part?**

No. Taking part is entirely voluntary but if you do decide to take part you can withdraw from the study at any time. If you decide to withdraw during the course of the study any data collected will be retained and may be used in an anonymised form for reports, presentations and publications. If you decide to participate, you will be asked to give consent.

**Are there benefits to me in taking part in this study?**

Whilst there are no immediate benefits for those people participating in the project, sharing your experiences and ideas will help us to develop a guidance document on recording harms in behavioural change intervention trials and any resulting recommendations for change.

**Are there any risks of taking part in this study?**

We do not anticipate any risks associated with taking part in this study.

**Will what I say be confidential?**

All the information that we collect about you during the course of the research will be kept strictly confidential and will only be accessible to members of the research team. You will not be able to be identified in any reports or publications unless you have given your explicit consent for this. If you agree to us sharing the information you provide with other researchers (e.g. by making it available in a data archive) then your personal details will not be included unless you explicitly request this. Electronic data will be password-protected and saved on the University of Sheffield’s server.

All participants will be reminded not to repeat what is discussed to others outside the group.

The research team will use your name, email, and telephone number to contact you about the research study. The only people in the University of Sheffield who will have access to information that identifies you will be the research team who need to contact you to arrange for the interview. Your contact details will be deleted as soon as possible after the interview has taken place and are no longer needed.

According to data protection legislation, we are required to inform you that the legal basis we are applying in order to process your personal data is that ‘processing is necessary for the performance of a task carried out in the public interest’ (Article 6(1)(e)). Further information can be found in the University’s Privacy Notice <https://www.sheffield.ac.uk/govern/data-protection/privacy/general>.

**What will happen to the data collected, and the results of the study?**

The interview will be audio recorded using encrypted Dictaphones. The content of the recordings will not be shared outside the research team.  Consent forms will be securely stored for 10 years and then all hard and electronic copies destroyed. The audio recordings will also be securely stored on university network drives and accessible only to the project team for the duration of the project.

University of Sheffield will keep non-identifiable information about you for ten years after the study has finished.

The University of Sheffield will act as Data Controller for this study. This means that the University is responsible for looking after your information and using it properly.

Once the interviews have been transcribed and analysed, the results will be used to assist in the development of a guidance document to advise Clinical Trials Units on approaches of recording harms in behavioural change intervention trials. Findings will be published in academic journals and presented in conferences, seminars, and workshops. Quotes from interviews may be used but will be anonymised.

**What are your choices about how your information is used?**

You can stop being part of the study at any time, without giving a reason, but we will keep information about you that we already have. We need to manage your records in specific ways for the research to be reliable. This means that we won’t be able to let you see or change the data we hold about you. If you agree to take part in this study, you will have the option for your data saved from this study to be used in future research.

**Who is organising and funding the study?**

The study is being led by researchers from the Clinical Trials Research Unit, School of Health and Related Research, University of Sheffield and funded by the National Institute for Health Research Clinical Trials Unit Support Funding.

**Who has reviewed the study?**

The study has been reviewed and approved by the University of Sheffield Research Ethics Committee (ref: 044669).

**What do I do if I have a concern about the conduct of this study?**

If you are dissatisfied with any aspect of the research and wish to make a complaint, please contact Diana Papaioannou, d.papaioannou@sheffield.ac.uk in the first instance. If you feel your complaint has not been handled in a satisfactory way you can contact the Head of the Department of the School of Health and Related Research (ScHARR): Professor Mark Strong, m.strong@sheffield.ac.uk. If the complaint relates to how your personal data has been handled, you can find information about how to raise a complaint in the University’s Privacy Notice: <https://www.sheffield.ac.uk/govern/data-protection/privacy/general>.

If you wish to make a report of a concern or incident relating to potential exploitation, abuse or harm resulting from your involvement in this project, please contact the project’s Designated Safeguarding Contact [Diana Papaioannou, d.papaioannou@sheffield.ac.uk]. If the concern or incident relates to the Designated Safeguarding Contact, or if you feel a report you have made to this Contact has not been handled in a satisfactory way, please contact the Head of the Department of School of Heath and Related Research: Professor Mark Strong, m.strong@sheffield.ac.uk, and/or the University’s Research Ethics & Integrity Manager (Lindsay Unwin; l.v.unwin@sheffield.ac.uk).

**Where can I get more information?**

If you have any questions, please contact:

- Diana Papaioannou, Principal Investigator. Email: d.papaioannou@sheffield.ac.uk
- Cara Mooney, Project Manager. Email: c.d.mooney@sheffield.ac.uk

Thank you for reading this information.

You will be given a copy of this information sheet and a signed consent form to keep.
