# Supplementary material for: Recording harms in randomised controlled trials of behaviour change interventions: a qualitative study of UK clinical trials units and NIHR trial investigators
Source: Trials. 2024 Mar 4;25:163. doi: 10.1186/s13063-024-07978-1 (PMC10910772; doi:10.1186/s13063-024-07978-1)
Supplement: Supplementary file 2 — Additional file 2. Informed Consent form. This file provides informed consent form used for interview participants. It was adapted for the participants taking part in the focus groups. [file 13063_2024_7978_MOESM2_ESM.docx]

*Additional File 2 provides the Informed consent form used in the individual interviews in the qualitative study as part of the RHABIT project. These files were modified for the focus groups conducted.*

**Principal Investigator**

Diana Papaioannou

[D.Papaioannou@sheffield.ac.uk](mailto:D.Papaioannou@sheffield.ac.uk)

**Co-investigators**

Cara Mooney

[c.d.mooney@sheffield.ac.uk](mailto:c.d.mooney@sheffield.ac.uk)

**Informed Consent Form**

**Individual Interview**

**Study title: Recording Harms in Behavioural change Intervention Trials**

**Initial each box**

1. I confirm that I have read and understand the Participant Information Sheet (version 2.0) dated 21/01/2022 for the above study. I have had the opportunity to consider the information, ask questions and have had these answered satisfactorily.
2. I understand that my participation is voluntary and that I am free to withdraw at any time. I do not have to give any reasons for why I no longer want to take part and there will be no adverse consequence if I choose to withdraw.
3. I understand and give permission for the interview to be audio recorded and for the resulting data to be used in reports and publications including anonymous direct quotation.
4. I understand and agree that my words may be quoted in publications, reports, web pages, and other research outputs. I understand that I will not be named in these outputs unless I specifically request this.
5. I understand that data collected will be stored securely, in accordance with the General Data Protection Regulation 2018 (GDPR) and the Data Protection Act 2018 which controls how your personal information is used by any organisation.
6. I understand that I am not obliged to answer any question in the interview.
7. I understand that the data collected from me in this study will be preserved and made available in anonymised form, so that they can be consulted and re-used by others.

1. I agree to take part in the above study, which has been subject to ethical reviews according to the procedures specified by the University of Sheffield Research Ethics Committee (ref: 044669).

**Participant details**

Name of Participant: ­­­­**_______________________________________**

Signature: Date:

**Researcher details**

Name of researcher taking consent:

Signature: Date:

**Project contact details for further information:**

**Principal Investigator**

Diana Papaioannou, d.papaioannou@sheffield.ac.uk

**Project Manager/Oversight**

Cara Mooney, c.d.mooney@sheffield.ac.uk

**The University of Sheffield**

Sheffield

S10 2TN
