# Supplementary material for: Recording harms in randomised controlled trials of behaviour change interventions: a qualitative study of UK clinical trials units and NIHR trial investigators
Source: Trials. 2024 Mar 4;25:163. doi: 10.1186/s13063-024-07978-1 (PMC10910772; doi:10.1186/s13063-024-07978-1)
Supplement: Supplementary file 3 — Additional file 3. Topic guide. This file provides the topic guide used in the interviews and focus groups. [file 13063_2024_7978_MOESM3_ESM.docx]

***RHABIT Study – recording harms/adverse events in behavioural change trials***

***Interview/Focus group Topic Guide V1.0 15Dec2021***

| - Had the information sheet - explained the project and also a document providing some information on existing literature and ideas on recording harms in these types of trials (BCT) - 30-40 minutes - Audio recording - Cover three main topics, your experience of recording and assessing harms in BCTs, your understanding and experience of using any models and looking back whether you would do anything differently next time. - Hopefully that all sounds OK and if you’re happy we’ll make a start. |
| --- |

***Topic 1 – Identification and assessment of harms….***

- - Maybe if we start with what your experience has been of recording harms in BCI trials, were AEs considered?
  - Were you expecting the intervention to cause a particular harm?
  - What factors were considered in deciding what AEs to record? E.g., population/ the intervention and resources, Sponsor processes.
  - Did you find any unintended harms/consequences, e.g., risk compensation, rebound effects, negative labelling?
  - Was there a perception that harms weren’t possible?

1. Who was involved in the decision process on what or how to record harms?
   - Sponsors, CIs, TSCs, DMCs etc. Did they actively participate/did they change your approach?

3. Did you record all AEs or selected AEs?

- - What informed this decision – did you or the team have a default position, or any standard definitions used, e.g. ICH GCP, only recording serious harms for example (need to know how serious harms were defined)
  - Why was this approach taken, did you find it useful?

1. How did you assess the AEs?
   - Determining what events might be expected in an intervention or population.
   - Relatedness
   - Expectedness for SUSARs

**Topic 2 – Recording harms…..**

1. How were harms recorded in your trial?
   - How well did this work?
   - Documented, database, etc.
   - Time and resources required to set-up and implement and processes
2. Who did the recording?
   - Site staff or research staff? (Or joint approach)
   - How did this work in practice?
   - Were there any issues?
3. What difference did the collection of AE data make to the trial overall?

- How was the AE data used?
- How did the data impact on the trial outcome/findings?

1. Qualitative sub studies/process evaluations nested in trials.

- If not discussed already, prompt as to whether these elements were included in trials and if they uncovered harms data
- Perceptions of staff as to importance of qualitative studies within trials on harms recording (i.e. not event level data)

**Topic 3 – Knowledge of existing models/typologies or mechanisms of harm from behaviour change…**

1. Were you aware of any of these models/literature?
   - If not, what were your first impressions of these principles for AE recording?
   - Any model preference and why
   - Implementation issues, e.g. resources, knowledge, understanding
2. Have you used any of these models?
   - If no, why not?
   - How confident would you feel in using these kinds of models?
   - How would you apply them?
   - What issues do you anticipate if you used these, if any?
   - If yes, how successful was it? / How well did they work?
   - Would you use this or another model again or in the future? (Prompt for reasons why/why not)
3. How important is this literature to you?

**Topic: 4 – Key considerations for recording harms in BCI trials….**

1. Looking back at your BCI trial(s) and having seen a bit on the literature, would you do anything differently next time?
   1. What would you tell other researchers?
