# Supplementary material for: Recording harms in randomised controlled trials of behaviour change interventions: a qualitative study of UK clinical trials units and NIHR trial investigators
Source: Trials. 2024 Mar 4;25:163. doi: 10.1186/s13063-024-07978-1 (PMC10910772; doi:10.1186/s13063-024-07978-1)
Supplement: Supplementary file 5 — Additional file 5. Coding tree-framework. This file provides the final coding tree/framework which was applied to the data in the transcripts. [file 13063_2024_7978_MOESM5_ESM.docx]

|  | **RHABIT - FRAMEWORK Development V0.2_24 Jun 2022** | |
| --- | --- | --- |
|  |  |  |
| 1 | **Factors that might influence considerations for collecting/ reporting potential harms in BCI's** | Population/condition - acceptability, age, sex, culture, religion etc/what is expected for this population |
|  |  | Plausibility vs just life events (where to draw the line) |
|  |  | Intervention (established or novel - risk) |
|  |  | Institutional v individual perspectives (professional backgrounds) |
|  |  | Study outcomes |
|  |  | Purpose of collecting/reporting |
|  |  | Liability |
|  |  | Consulting with OR impact on - significant others |
|  |  |  |
| 2 | **Perception of harms in BCIs** | Low risk - can BCI/social interventions do harm (not always positive) |
|  |  | Fear of missing harms if don't look for everything |
|  |  | Perception of risk informed by knowledge and background of the individual |
|  |  | Risk can change within participants and depending on context |
|  |  | Complex intervention equals complex harms |
|  |  | Need to recognise different types of harm e.g. result of negligence/result of intervention |
|  |  |  |
| 3 | **Currently used approaches to recording harms in BCI trials** | Augment default SAE reporting with bespoke measures to detect events - needed due to complexities of BCI |
|  |  | Case-by-case context driven decision process and bespoke management strategies - assumptions based on poor info, can you assign causality with confidence |
|  |  | Process of considering harms is more implicit, not explicit or structured |
|  |  | Standard ICH-GCP SAE reporting terminology and process used (recognisable/respected, confidence in the system, easier to use than not, lack of alternative) |
|  |  | Protocol theoretically should justify approach and rationale for recording/not recording events - sporadic in BCI's |
|  |  | Plausibility - use a logical/common sense approach when making decisions |
|  |  | Rarely set pre-specified events - Difficult to set predefined list of expected harms as populations & interventions/therapies are complex, & conditions may change naturally/fluctuate |
|  |  | Outcomes driven - looking for paradoxical effects/treatment failure |
|  |  | Priming participants and staff - create a harm where there wasn't one |
|  |  | Trial risk assessment to help inform |
|  |  | Use number of events to inform decision on plausibility and reporting |
|  |  | QA's preference to use default approach |
|  |  |  |
| 4 | **Assessment (relatedness etc)** | How to quantify harm in BCI's |
|  |  | Uncertainty how to assess relatedness |
|  |  | Social discussion to make decision - not statistical |
|  |  | Attribution (relatedness) role - DMC |
|  |  | Need for detailed information to make a decision not always easily available - more complex |
|  |  | Clinician judgment/decision, rarely non-clinician - knows the patient in context |
|  |  | Judgment based on perception of harm |
|  |  | Follow standard SOPs for expedited process (SAEs) |
|  |  |  |
| 5 | **Logistics and practicalities** |  |
|  | **WHO** | Multidisciplinary clinical and academic experience essential (including CIs, clinicians, trial management, statisticians) |
|  |  | Those collecting data - facilitators, assessors etc - Need for training data collection staff as no specialists |
|  |  | PPI input essential - events should be important and meaningful |
|  |  | Validation role of sponsor, QA, ethics, oversight committees (TSC/DMC) |
|  |  | Strongest voice can drive decision making |
|  |  | CTUs as 'objective' voice/challenge models |
|  |  | Responsibility for decision making is unclear - CI or TM |
|  |  | Dependent on the trial - case-by-case basis |
|  |  |  |
|  | **WHAT** | Proportionate approach (benefit v burden) - Data collected needs to be relevant to safety, analysis and reporting |
|  |  | Meaningful to participants/patients - PPI Involvement |
|  |  | Meaningful to research - loose core data if collecting too much/skew data/noise |
|  |  | Everything for lack of better guidance/knowledge/fear of missing data |
|  |  | Informed by burden of data collection |
|  |  | Dependent on type of trial & its outcomes |
|  |  | Outcomes/ endpoints may cover AEs |
|  |  | Perception SAEs/causality easier to identify than AEs |
|  |  | Quantitative data/statistics most useful for DMEC - decision making |
|  |  | Typically context/individual driven - more information required for decision making (can be difficult to get this level of info) - judgement is subjective not scientific |
|  |  | Harms at point of screening, randomisation, intervention and follow-up |
|  |  |  |
|  | **HOW** | Embedded qualitative work as an adjunct to identify harms/flag events not covered by quantitative measures |
|  |  | Need a structured/more efficient way to talk about potentially harmful mechanisms as part of interviews |
|  |  | Challenge - without interrupting the intervention/becoming part of the intervention) |
|  |  | Qualitative does not identify causal attribution |
|  |  | Better at teasing out nuances than questionnaires |
|  |  | Recorded in the CRF/data collected via standard or bespoke questionnaires |
|  |  | Purpose designed data collection templates, validated questionnaires, standard SAE forms |
|  |  | Standardised measures are always sensible & useful |
|  |  | Tolerability, adherence, compliance |
|  |  | Issue of old & outdated measures |
|  |  | Best practice - Validated questionnaires? (Sponsor viewpoint) |
|  |  | Burden on participants - Finite amount of times can contact participants on sensitive info |
|  |  |  |
| 6 | **Resources** | Time and resources required dependent on the trial and what data is being collected - more data, more resources required |
|  |  | Trial should be adequately funded to support data collection and reporting |
|  |  | Potential burden on participants and/or staff (physically and emotionally) |
|  |  | Can be wasted effort/resources - under or over reporting |
|  |  |  |
| 7 | **How was event data used** | Reporting safety in journal publications/dissemination - although better consistency needed in BCI trials |
|  |  | Event data required as standard to publish in some journals |
|  |  | DMC/TSC oversight committee reports - reliant on data accuracy to make trial decisions |
|  |  | Not all data collected used/analysed - waste of resource |
|  |  | Safeguarding only - not reported |
|  |  | Ethical to report safety data |
|  |  | Part of monitoring safety |
|  |  | Effectiveness of the intervention/between group comparison |
|  |  |  |
| 8 | **Awareness/implementation of existing models/typologies and frameworks** | Overall lack of awareness of literature - but found the summary helpful and would use in future work |
|  |  | Would need training/experienced input how to use models |
|  |  | Used model to theorise harms for BCIs (intervention development/feasibility) - psychological imagination |
|  |  | Use of CONSORT extension to guide reporting of AEs |
|  |  |  |
|  | **Experience of methods used (no knowledge of existing models)** | Literature searching within and outside comparable studies and conditions |
|  |  | Theorised potential harms writing the protocol |
|  |  |  |
|  | **Future working** | Team should read about existing models in preparation for AE discussion |
|  |  | Models provide considerations what to think about when designing and setting up BCI trials |
|  |  | Approach will need to be sensitive to complexities of BCI trials |
|  |  | More direction (structure and process) required from authorities e.g. Funders, HRA, MHRA |
|  |  | Guidance/signposting needed, but also practical application (latter challenging considering complexity and diversity of BCI trials) |
|  |  | Protocol templates should include wording/advice based on contractual & regulation obligations |
|  |  | Literature should be accessible and concise (lack of time) |
|  |  | Need for consistency |
|  |  | Bespoke trial specific staff training |
|  |  | Data collection should be proportionate - not to burden participants or staff |
|  |  | Intervention development and feasibility should more directly identify potential unintended harms e.g. through qualitative work (although small scale and more resources needed at this stage to do this) |
|  |  | Can psychological harms go beyond the end of a trial? |
|  |  | Look at potential of indirect harm on others, i.e. family members/friends of participant |
|  |  |  |
| 9 | **Should we record events in BCI trials** | It is important to consider and report events/harms in BCI trials - you are 'injecting a thought process' - how to measure this |
|  |  | Part of establishing merits of an intervention |
|  |  | Need for transparency but events now often clear cut |
|  |  | Its about patient safety - issue is not should but what, when and how |
|  |  | Focus on intervention benefits, not potential for harms - need to be more objective |
|  |  | Fear of consequences if do not record e.g. accessing funding, repercussions for researchers and CTUs |
|  |  |  |
| 10 | **Language/terminology** | Current terminology is medicalised and pharma based (Mismatch) - obliged to use this as no alternative |
|  |  | Used in line with existing SOPs |
|  |  | Need for a definition/examples of AEs in CBI's as unclear (what is the difference between an SAE, AE, Safeguarding and compliance in BCIs) |
|  |  | Are they AEs or 'incidents of concern'? |
|  |  | Hard to define, when does it become an AE? |
|  |  | What is a harm is subjective - dependent on the individual |
